# WaveAttention-ResNet: a deep learning-based intelligent diagnostic model for the auxiliary diagnosis of multiple retinal diseases

**DOI:** 10.3389/fradi.2025.1608052

**Published:** 2025-07-29

**Authors:** Biao Guo, Daqing Wang, Ruiqi Zhang, Jia Hou, Wenchao Liu, YongFei Wu, Xudong Yang, Lijuan Zhang

**Affiliations:** ^1^ Netchina Huaxin Technology Co., Ltd, Taiyuan, Shanxi, China; ^2^ Shanxi Eye Hospital, Taiyuan, Shanxi, China; ^3^ Department of Vitreoretinal, Shanxi Eye Hospital, Shanxi Medical University, Taiyuan, Shanxi, China; ^4^ College of Artificial Intelligence, Taiyuan University of Technology, Taiyuan, Shanxi, China

**Keywords:** deep learning, retinal diseases, ResNet18, CBAM, wavelet convolution

## Abstract

**Objective:**

This study constructs a deep learning-based combined algorithm named WaveAttention ResNet (WARN) to investigate the classification accuracy for seven common retinal diseases and the feasibility of AI-assisted diagnosis in this field.

**Methods:**

First, a deep learning-based classification network is constructed. The network is built upon ResNet18, integrated with the Convolutional Block Attention Module (CBAM) and wavelet convolution modules, forming the WARN method for retinal disease classification. Second, the public OCTDL dataset is used to train WARN, which contains classification data for seven retinal disease types: age-related macular degeneration (AMD), diabetic macular edema (DME), epiretinal membrane (ERM), normal (NO), retinal artery occlusion (RAO), retinal vein occlusion (RVO), and vitreomacular interface disease (VID). During this process, ablation experiments and significance tests are conducted on WARN, and comprehensive analyses of various indicators for WARN, ResNet-18, ResNet-50, Swin Transformer v2, EfficientNet, and Vision Transformer (ViT) are performed in retinal disease classification tasks. Finally, data provided by Shanxi Eye Hospital are used for testing, and classification results are analyzed.

**Results:**

WARN demonstrates excellent performance on the public OCTDL dataset. Ablation experiments and significance tests confirm the effectiveness of WARN, achieving an accuracy of 90.68%, F1-score of 91.29%, AUC of 97.50%, precision of 93.31%, and recall of 90.68% with relatively short training time. In the dataset from Shanxi Eye Hospital, WARN also performs well, with a recall of 90.85%, precision of 79.94%, and accuracy of 89.18%.

**Conclusion:**

This study fully confirms that the constructed WARN is efficient and feasible for classifying seven common retinal diseases. It further highlights the enormous potential and broad application prospects of AI technology in the field of auxiliary medical diagnosis.

## 1 Introduction

The accelerating global aging process has made retinal diseases a significant public health issue threatening visual health. Age-related macular degeneration (AMD) has a global prevalence of approximately 8.7%, serving as the primary cause of blindness among individuals over 60 years old in developed countries (1). Diabetic retinopathy affects about 22.27% of diabetic patients, emerging as the main factor for visual impairment in working-age populations (2). Diabetic macular edema (DME), the leading cause of vision loss in diabetic patients, impacts approximately 5.5% of this population (3). Retinal vein occlusion (RVO) has a prevalence of about 0.5% in individuals aged 30 and above, with over 16 million affected people worldwide (4). These diseases are highly disabling, often leading to irreversible vision damage in advanced stages and exacerbating socioeconomic burdens (5).

Optical coherence tomography (OCT) is regarded as the “gold standard” for diagnosing retinal diseases such as AMD (age-related macular degeneration) (6–8), DME (diabetic macular edema) (9–12), and ERM (epiretinal membrane) (13–15). In contrast, fundus photography has certain limitations in diagnosing these conditions. As a non-invasive imaging technique, OCT has become an essential tool for retinal disease diagnosis due to its high resolution, rapid imaging, and good safety. However, the interpretation of OCT images currently relies mainly on manual reading, suffering from issues such as long time consumption and significant subjectivity differences. Additionally, the obvious imbalance between the number of ophthalmologists and the growing demand for examinations further causes diagnostic delays, affecting timely patient treatment. Therefore, developing artificial intelligence (AI)-based auxiliary diagnostic systems is of great significance for improving diagnostic efficiency and consistency and alleviating clinical resource shortages (16).

AI applications in medical image analysis have achieved remarkable progress. In the field of ophthalmology, AI processes complex medical images through deep learning techniques, excavating hidden information to provide comprehensive diagnostic evidence (17, 18). Early studies, such as Xu et al., proposed an improved method for classifying retinal arterioles and venules, providing tools for early disease diagnosis (19). Najeeb et al. used a single-layer CNN to achieve region-of-interest detection in retinal OCT scans (20). With technological advancements, Gao et al. trained a deep CNN with an 88.72% accuracy in grading the severity of diabetic retinopathy (21). Fang et al. proposed a lesion-aware convolutional neural network (LACNN) that simulates the diagnostic thinking of ophthalmologists, enhancing classification efficiency (22).

Despite these advancements, existing studies still have the following limitations:

(1) Most models are based on single-modal data, lacking integration of multi-modal information;
(2) Insufficient classification capabilities for rare diseases or images with multiple lesions;
(3) Limited applicability in clinical environments with constrained computational resources, as model training is time-consuming and updates are restricted.

This study proposes the WaveAttention-ResNet (WARN) model, which enhances feature extraction capabilities by integrating the CBAM attention mechanism and wavelet convolution modules. The goal is to improve the classification accuracy and training performance for seven common retinal diseases, providing a new approach for the widespread clinical application of AI-assisted diagnosis.

## 2 Methods

### 2.1 Network

We have successfully constructed a classification method for retinal diseases, namely the wavelet-enhanced ResNet (WaveAttention-ResNet, WARN) based on the Convolutional Block Attention Module (CBAM).

During the process of building this method, we first selected ResNet-18 as the basic framework. The reason for this choice is mainly that ResNet-18 has a concise and efficient structure. This structure can not only effectively avoid the gradient vanishing problem that often occurs in deep networks but also is easy to expand and modify in practical applications, providing great convenience for subsequent optimization work.

In order to further enhance the discriminative ability of the model and enable it to more accurately identify features in the analysis of complex retinal disease images, we skillfully added the CBAM module after the wavelet convolution. The working principle of the CBAM module is to perform attention weighting on the two dimensions of channels and space, enabling the model to focus more on those important features. In this way, it can effectively reduce the interference of background noise on the judgment results and significantly improve the accuracy and reliability of the model analysis.

However, with the increasing complexity of the retinal disease classification task, we found that traditional convolutional operations may not be able to fully capture all the detailed information in the images in some cases. In view of this, we introduced a wavelet convolution module after the CBAM module. The purpose of introducing this module is to make full use of the unique advantages of wavelet transform to further enhance the feature extraction process. Specifically, wavelet transform allows us to conduct a detailed analysis of the images at different scales, so as to better capture the local details and global structures in the images, enabling the model to have a more comprehensive and in-depth understanding of the image information.

**Figure 1:**
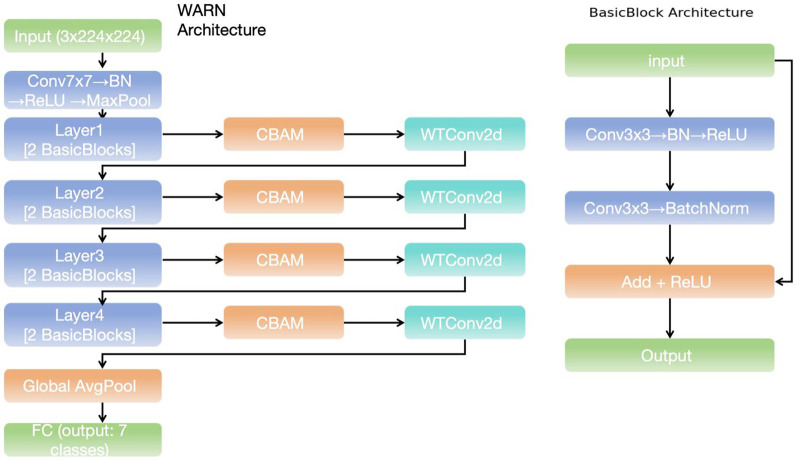
WARN architecture.

As shown in Figure 1, the network we constructed is a deep learning model of wavelet-enhanced ResNet based on CBAM. This model significantly improves the feature extraction ability and the utilization efficiency of spatial-channel information by organically combining the CBAM attention mechanism and the wavelet transform convolution (WTConv2d) module. From the perspective of the overall architecture, this network is composed of multiple ResNet modules, CBAM modules, and WTConv2d modules. Its design concept is to achieve the classification task of retinal disease images efficiently through multi-level feature extraction and enhancement, providing powerful technical support for the accurate diagnosis of retinal diseases.

#### 2.1.1 ResNet-18

ResNet-18, as a classic deep convolutional neural network, was first proposed in 2015 (23). During the training of deep networks, the problems of gradient vanishing and degradation are common and urgent challenges to be solved. ResNet-18 successfully overcame this problem by introducing the Residual Learning mechanism.

The core highlight of ResNet-18 lies in its unique skip connection design. This ingenious design allows the information in the network to be directly transferred from one layer to another without being interfered by non-linear transformations at all. It is precisely because of this design that the network can effectively learn the identity mapping, enabling the successful implementation of deeper networks.

In terms of the overall architecture, ResNet-18 is mainly composed of five parts. Firstly, there is the input layer, which contains a 7 × 7 convolutional layer with a stride of 2. The main function of this convolutional layer is to extract the basic features of the image, and it further reduces the size of the feature map through a max pooling layer.

Following the input layer are four core stages (Stage), each of which is composed of multiple residual blocks, and these residual blocks are exactly the key components of ResNet-18. Inside each residual block, the input data will be processed through two paths respectively. The main path contains two 3 × 3 convolutional layers, and each convolutional layer is followed by a Batch Normalization (BN) operation and a ReLU activation function; the auxiliary path, that is, the skip connection, serves to directly add the input to the output. When the dimensions of the input and output are the same, this operation is very straightforward; however, if the dimensions are different, a 1 × 1 convolution is required to adjust the input dimension to match the output dimension. As the network layers go deeper, the spatial resolution of the feature map gradually decreases, while the number of channels gradually increases. Such changes are conducive to capturing more complex patterns.

After the last convolutional stage is completed, the Global Average Pooling (GAP) layer comes into play, which compresses each feature map into a scalar value. Through this operation, the number of parameters is significantly reduced, and the risk of overfitting is also decreased accordingly. Finally, the Fully Connected Layer maps these processed features into the specific category space, thus completing the entire classification task.

Considering the simplicity of the ResNet-18 structure, the convenience in its actual implementation, its relatively short training time, and its certain feature extraction ability, among other factors, we finally decided to choose ResNet-18 as the basic network architecture.

#### 2.1.2 CBAM

CBAM (Convolutional Block Attention Module) (24), as a unique attention mechanism, was originally designed to significantly enhance the feature representation ability in convolutional neural networks (CNNs).

The inspiration for the attention mechanism actually comes from the unique characteristics of the human visual system. As we know, when the human eye observes an image, it does not process the entire image uniformly and indifferently. Instead, according to specific task requirements, it selectively focuses on certain specific regions or features in the image. In the field of deep learning, ingeniously introducing the attention mechanism enables the model to be more sensitive and focused on the truly important parts of the input data, thereby effectively improving the overall performance of the model.

**Figure 2:**
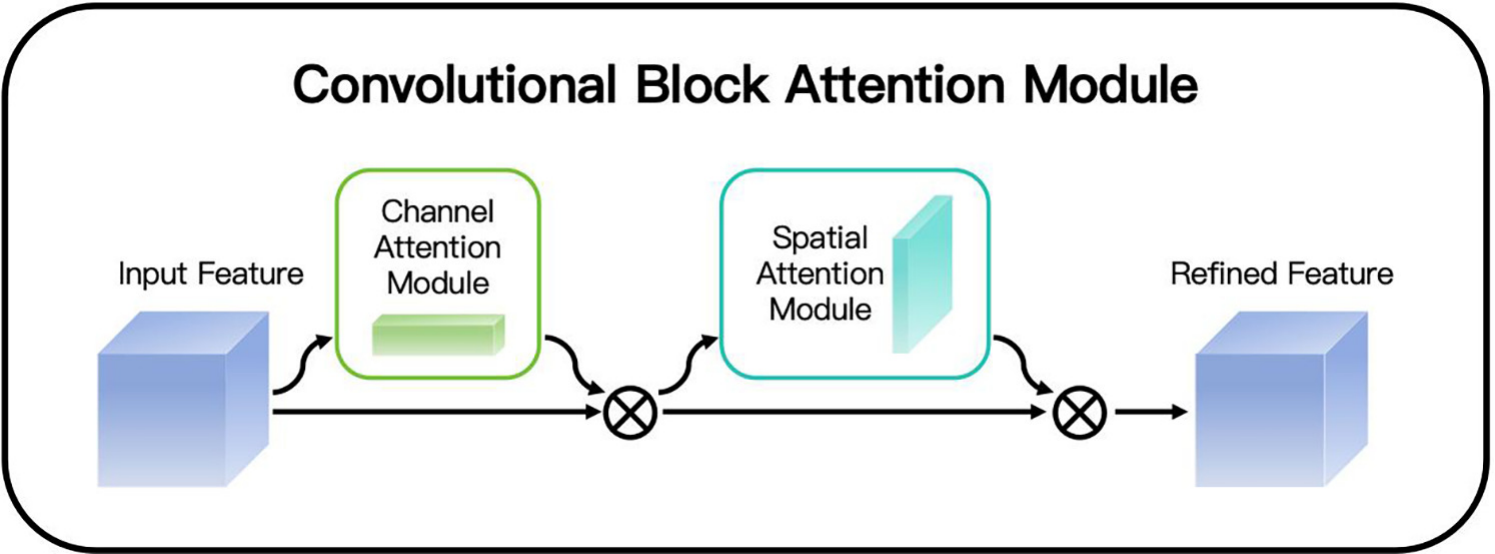
CBAM.

Looking at the working principle of CBAM in detail (as shown in Figure 2), it mainly consists of two key steps: first, Channel Attention, and second, Spatial Attention. The channel attention mechanism focuses on analyzing and weighting different channels of the feature map. By comprehensively considering the importance of the information contained in each channel, it assigns corresponding weights to different channels, enabling the model to highlight those channels that contain key information and thus enhancing the ability to capture important features. The spatial attention mechanism mainly focuses on the spatial dimension of the feature map. It analyzes each position of the feature map to determine which spatial positions contain information that is more crucial for the current task, and then assigns higher weights to these positions, enabling the model to more accurately focus on the important spatial regions in the image and further improving the understanding and expression ability of the image features. These two steps cooperate with each other and complement each other, jointly acting on the convolutional neural network, enabling the network to more efficiently extract and utilize key feature information when processing image data.

**Figure 3:**
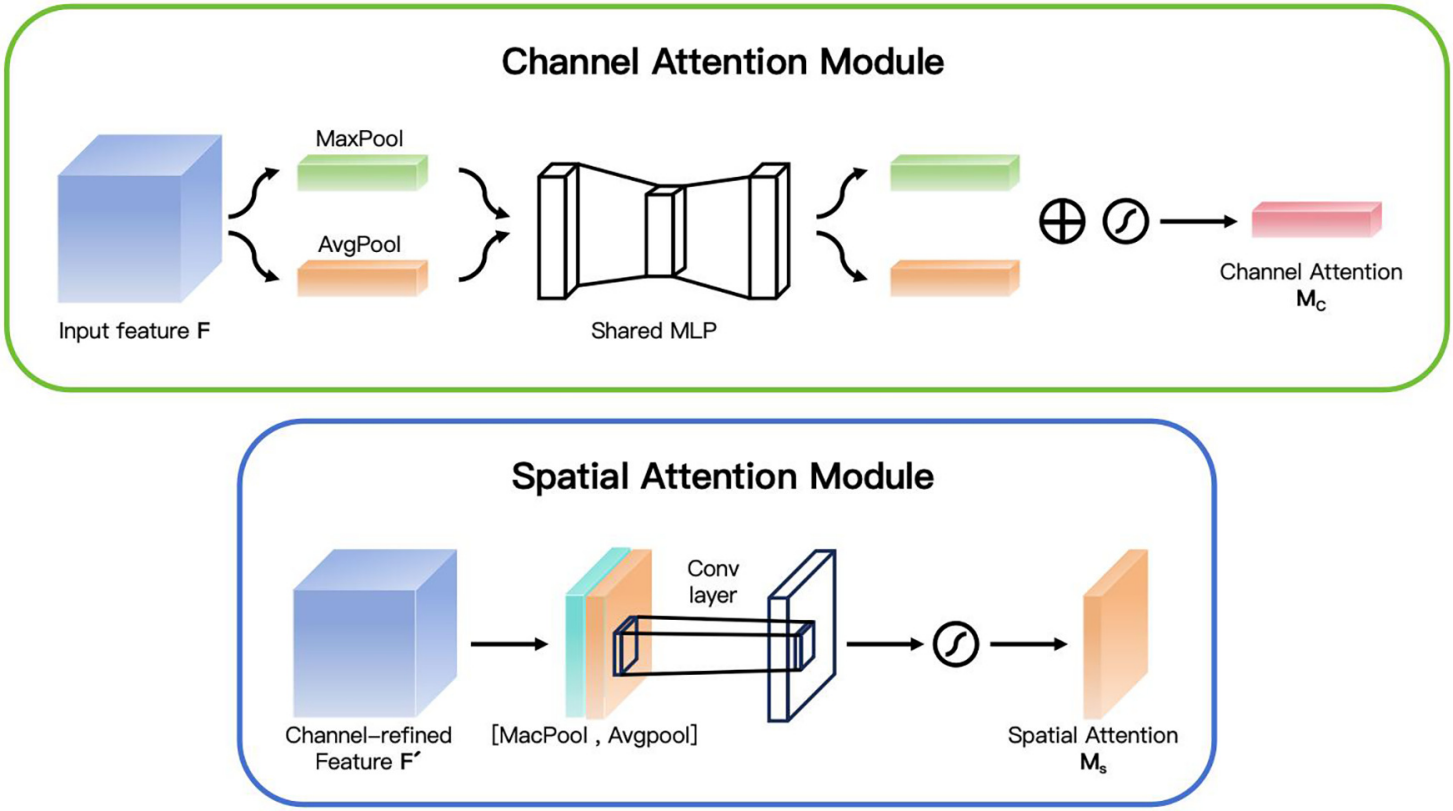
Channel attention and spatial attention.

In the Channel Attention stage, as shown in Figure 3, the feature maps of each channel are adjusted by calculating the importance of each channel. First, it calculates the results of the max pooling and the average pooling for each channel respectively. Then, a shared Multi-Layer Perceptron (MLP) network is used to calculate the weight of each channel. These weights reflect the importance of each channel. Finally, a Sigmoid function is used to transform these weights into values between 0 and 1, which are then multiplied by the original feature map to enhance or suppress the information of certain channels. The channel attention mechanism is used to highlight the most important channel information in the feature map and suppress the unimportant channels. Specifically, given the input feature map $X\in R^{C\times H\times W}$, we first generate two descriptors $F_{\mathrm{gap}}(X)$ and $F_{\mathrm{gmp}}(X)$ through the operations of Global Average Pooling (GAP) and Global Max Pooling (GMP). Both of these two descriptors are C-dimensional vectors. Then, we input these two descriptors into a Multilayer Perceptron (MLP), which consists of a hidden layer and a linear layer. Assuming that the dimension of the hidden layer is C/r (where r is the dimensionality reduction ratio), the output of the MLP can be expressed as:

$$M_{c}(X)=\sigma (\mathrm{MLP}(\left[F_{\mathrm{gap}}(X);F_{\mathrm{gmp}}(X)\right]))$$

where the symbol *σ* represents the Sigmoid activation function. Finally, the channel attention weights $M_{c}(X)$ are applied to the input feature map X through element-wise multiplication:

$$X{}^{\prime }=X\cdot M_{c}(X)$$

In the Spatial Attention stage, as shown in Figure 3, CBAM further calculates the importance of each spatial position. It performs max pooling and average pooling operations on the feature map output by the channel attention module, and then combines these two results through a convolutional layer to finally obtain a spatial attention map. This attention map is also transformed by a Sigmoid function and then applied to the original feature map to strengthen or weaken the features at different spatial positions. The spatial attention mechanism focuses on highlighting the most important spatial regions in the image. Given the feature map *X*' processed by the channel attention, we first perform max pooling and average pooling operations on each channel to generate two H×W feature maps $F_{\max}(X{}^{\prime })$ and $F_{\mathrm{avg}}(X{}^{\prime })$. Then, we concatenate these two feature maps together, and generate the spatial attention weights through a 7 × 7 convolutional layer and a Sigmoid activation function.

$$M_{s}(X{}^{\prime })=\sigma (\mathrm{Con}v_{7}(\left[F_{\max}(X{}^{\prime });F_{\mathrm{avg}}(X{}^{\prime })\right]))$$

The final spatially attention-weighted feature map *X*'’ can be expressed as:

$$X{}^{\text{″}}=X{}^{\prime }\cdot M_{s}(X{}^{\prime })$$

This combination method enables the model to pay more attention to the important regions in the image, while suppressing the background noise and other irrelevant details. When dealing with OCT images, CBAM enhances the model's ability to capture key features by emphasizing them. Specifically, CBAM first evaluates the importance of each feature channel through the channel attention mechanism and assigns higher weights to the more important channels, thereby highlighting the information that is crucial for diagnosis. Secondly, the spatial attention mechanism focuses on identifying which regions in the image are most critical for diagnosis and strengthens the information in these regions, enabling the algorithm to more accurately locate the lesion positions.

#### 2.1.3 Wavelet convolution module

**Figure 4:**
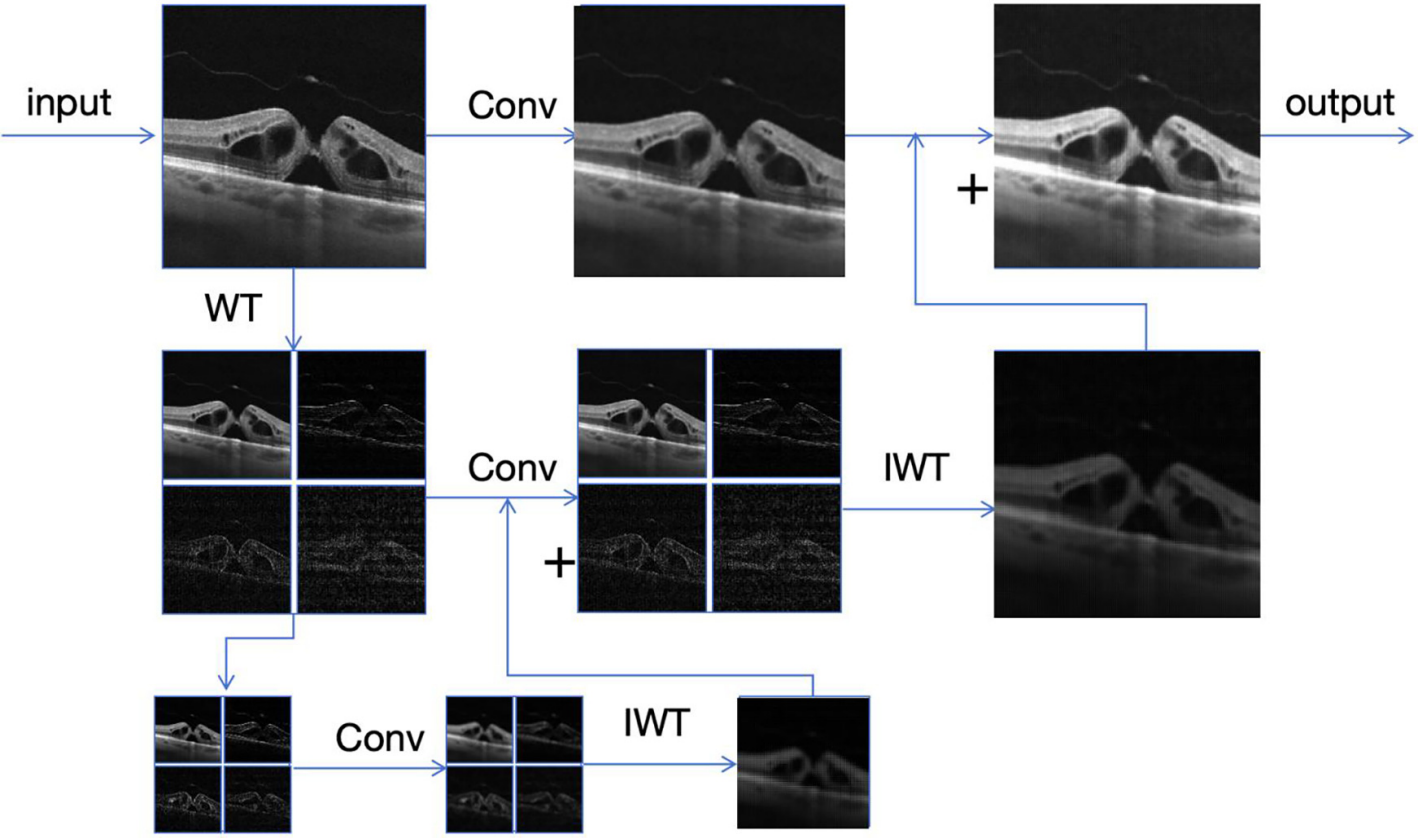
Wavelet convolution module.

As shown in Figure 4, the core of the wavelet convolution module (25) lies in using wavelet transform to decompose the input feature map into low-frequency (LL) and high-frequency (LH, HL, HH) sub-bands, which respectively represent the main structural information and edge or detail information of the image. This ability of multi-resolution analysis is difficult to achieve by traditional convolutional layers. For the decomposed sub-bands, we adopt the depth convolution operation with small kernels (3 × 3). This can not only effectively reduce the number of parameters but also maintain or even enhance the accuracy of feature extraction. Finally, these processed sub-bands are recombined into a complete output feature map through the inverse wavelet transform (IWT). This method not only reduces the computational cost but also improves the adaptability of the model to features at different scales.

The Wavelet Transform (WT) is a powerful signal processing tool that can decompose an input signal into different frequency components. For a two-dimensional image, we can use the Haar wavelet transform to decompose it into four parts: the low-frequency component $X_{\mathrm{LL}}$, the horizontal high-frequency component $X_{\mathrm{LH}}$, the vertical high-frequency component $X_{\mathrm{HL}}$, and the diagonal high-frequency component $X_{\mathrm{HH}}$. These components respectively correspond to different features of the input signal. The low-frequency component $X_{\mathrm{LL}}$ represents the main structural information of the signal, usually containing the overall shape and low-frequency trend of the image. The horizontal high-frequency component $X_{\mathrm{LH}}$ captures the edge or texture information in the horizontal direction, the vertical high-frequency component $X_{\mathrm{HL}}$ captures the edge or texture information in the vertical direction, and the diagonal high-frequency component $X_{\mathrm{HH}}$ captures the detail or noise information in the diagonal direction. By applying different types of filters (low-pass and high-pass), we can decompose the image into different frequency bands, thus achieving different hierarchical expressions of the image information. The specific formulas are as follows:

$$f_{\mathrm{LL}}=\frac{1}{2}\left[\begin{array}{cc} 1 & 1\\ 1 & 1 \end{array}\right],\;f_{\mathrm{LH}}=\frac{1}{2}\left[\begin{array}{cc} 1 & -1\\ 1 & -1 \end{array}\right],\;f_{\mathrm{LL}}=\frac{1}{2}\left[\begin{array}{cc} 1 & 1\\ -1 & -1 \end{array}\right],\;f_{\mathrm{LL}}=\frac{1}{2}\left[\begin{array}{cc} 1 & -1\\ -1 & 1 \end{array}\right]$$

By performing a convolution operation on the input feature map *X*" through the above filters and combining it with a downsampling operation with a stride of 2, we can obtain four subband feature maps:

$$\left[X_{\mathrm{LL}},\;X_{\mathrm{LH}},\;X_{\mathrm{HL}},\;X_{\mathrm{HH}}]=\mathrm{Conv}([\;f_{\mathrm{LL}},\;f_{\mathrm{LH}},\;f_{\mathrm{HL}},\;f_{\mathrm{HH}}],\;X^{\prime \prime }\right)$$

As shown in the figure above. Next, a small convolutional kernel (3 × 3) is applied to each subband feature map for feature extraction. Assuming that the weight tensor of the convolutional kernel is *W*, the convolution operation can be expressed as:

$$Y_{\mathrm{LL}},\;Y_{\mathrm{LH}},\;Y_{\mathrm{HL}},\;Y_{\mathrm{HH}}=\mathrm{Conv}(W,[X_{\mathrm{LL}},\;X_{\mathrm{LH}},\;X_{\mathrm{HL}},\;X_{\mathrm{HH}}])$$

Finally, these feature maps are reconstructed into a feature map of the original resolution through the Inverse Wavelet Transform (IWT):

$$Y=\mathrm{IWT}(Y_{\mathrm{LL}},\;Y_{\mathrm{LH}},\;Y_{\mathrm{HL}},\;Y_{\mathrm{HH}})$$

This design not only expands the receptive field of the model but also enables it to capture richer frequency information while maintaining a relatively low number of parameters, which helps to improve the classification accuracy.

This method transforms the input image into the wavelet domain, performs convolution operations on different frequency components, and reconstructs the output image using the inverse wavelet transform. Specifically, first apply the Haar wavelet transform to the input imageX, decomposing it into a low-frequency component $\;X_{\mathrm{LL}}$ and three high-frequency components $X_{\mathrm{LH}}$,$X_{\mathrm{HL}}$, and $X_{\mathrm{HH}}$. Subsequently, lightweight deep convolution operations with small kernels are applied to each frequency component. Then, the inverse wavelet transform (IWT) is used to fuse all convolved components to generate an intermediate output feature map. To preserve the structural information in the original image, the final output is obtained by adding the feature map after inverse wavelet transform to the convolved original input image. This process can be recursively applied to the low-frequency component $\;X_{\mathrm{LL}}$ to achieve multi-level wavelet decomposition, effectively expanding the receptive field without significantly increasing the number of parameters, while retaining image details and structural information.

### 2.2 Datasets

The newly released OCTDL (26) dataset was selected for this study, featuring remarkable characteristics. It comprehensively covers seven distinct retinal diseases, providing invaluable resources for the diagnosis and in-depth research of ophthalmic disorders.

Images in the dataset were sourced from multiple institutions, including Ural Federal University, Professor Plus Eye Surgery Clinic, and Ural State Medical University. This multi-institutional collaboration ensures the dataset's diversity and representativeness. All images were acquired using an Optovue Avanti RTVue XR device, which utilizes advanced scanning parameters (e.g., dynamic scanning length and high-resolution imaging) to guarantee the quality and detail richness of the collected data.

The annotation process was rigorous and multi-staged: first, seven medical students performed initial annotations; subsequently, two experienced clinical experts independently reviewed the annotations to minimize errors; finally, the clinic director confirmed the annotations to ensure accuracy and consistency.

**Figure 5:**
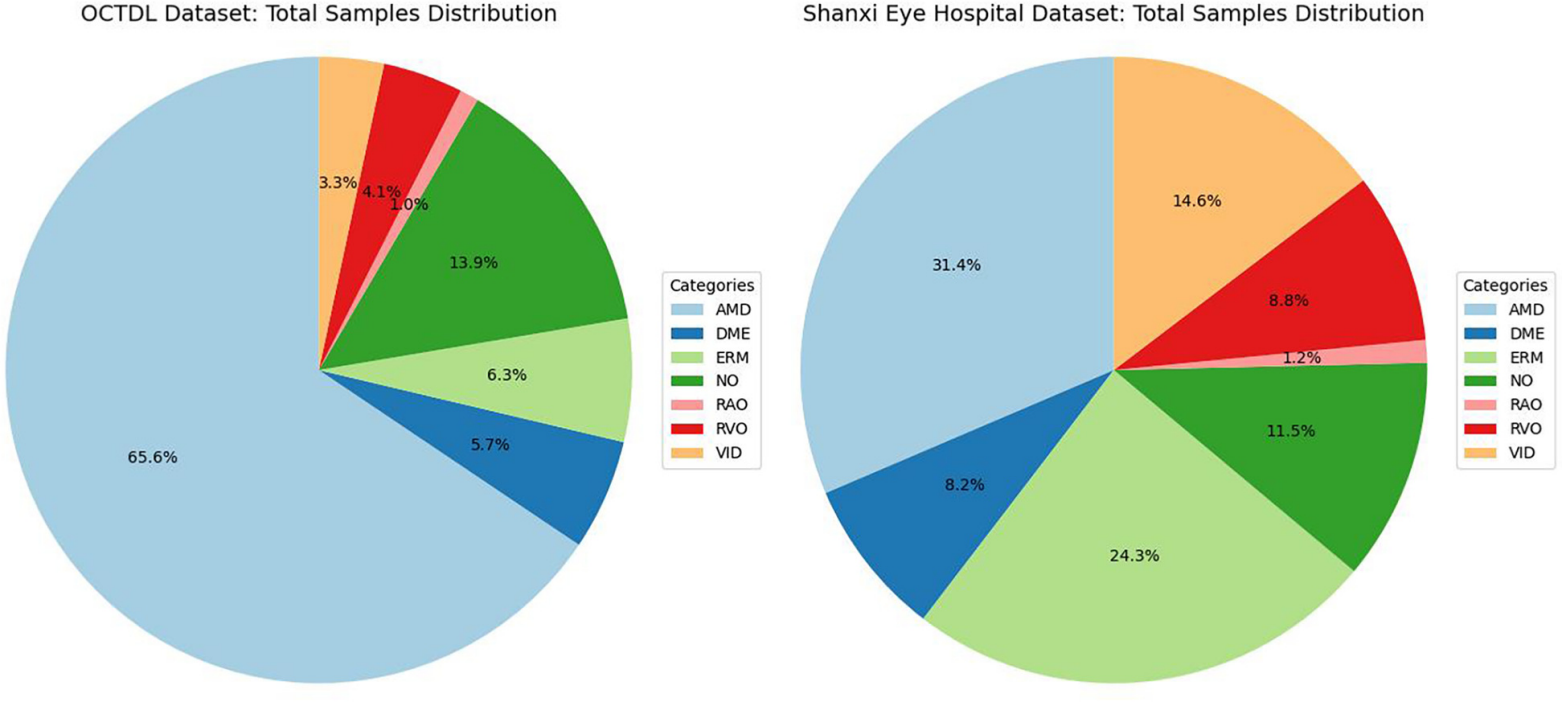
Dataset distribution.

Comprising 2,064 images from 821 patients (age range: 20–93 years, male-to-female ratio 3:2, mean age 63 years), the dataset covers seven fundus disease types: age-related macular degeneration (AMD), diabetic macular edema (DME), epiretinal membrane (ERM), normal (NO), retinal artery occlusion (RAO), retinal vein occlusion (RVO), and vitreomacular interface disease (VID). Figure 5 illustrates the dataset distribution.

Additionally, Shanxi Eye Hospital provided a pathological image dataset with OCT data acquired by two devices (Heideiberg; VG 200D, Intalight Ltd., China). The dataset includes AMD, DME, ERM, NO, RAO, RVO, and VID (encompassing macular holes and vitreomacular traction syndrome), matching the lesion types in the OCTDL dataset to facilitate comparative analysis.

Annotation was conducted by three experts with over five years of clinical experience in retina, with discrepancies resolved by a senior expert with 20+ years of experience. The 942 annotated images serve as valuable resources, and their distribution is shown in Figure 5.

### 2.3 Ethical approval

This study was approved by the Ethics Committee of Shanxi Eye Hospital (Approval No.: SXYYLL-KSSC021) and strictly adheres to the principles outlined in the Declaration of Helsinki. Given the retrospective nature of data collection and the use of de-identified images, informed consent was not required.

### 2.4 Experimental setup

In this experiment, all models were trained with uniform parameters: 100 epochs, batch size of 128, Adam optimizer, learning rate of 0.0001, weight decay of 0.0005, cosine annealing learning rate scheduler, and cross-entropy loss function. The experimental environment comprised an NVIDIA GeForce RTX 4060 GPU, 12th Gen Intel(R) Core(TM) i7-127000 2.10 GHz CPU, Python 3.12, and PyTorch 2.6.0.

### 2.5 Data processing

The OCTDL dataset was randomly split into training, validation, and test sets at a patient-level ratio of 60:10:30, ensuring that images from each patient belonged to only one subset. This approach prevented data cross-contamination, safeguarded the independence and fairness of model training, validation, and testing, and enhanced generalization to new patient data. To mitigate overfitting and improve model generalization, data augmentation was applied to the training set, including random cropping, horizontal/vertical flipping, color distortion, rotation, translation, and Gaussian blur. Each augmentation method was controlled by application probabilities with rational parameter ranges to avoid excessive distortion.

Additionally, addressing the class imbalance issue, a class-balanced sampling strategy was adopted with a sampling weight decay rate of 0.9. This enabled the model to prioritize minority classes in the early training phase and gradually align with the overall distribution in later stages, thereby enhancing overall classification performance.

### 2.6 Comparative experiment

To evaluate and compare the performance of different deep learning models in the classification of Optical Coherence Tomography (OCT) images, a series of experiments were designed for comparison, including the networks of ResNet-18, ResNet-50 (23), Swin Transformer v2 (27), EfficientNet (28), and Vision Transformer (ViT) (29).

ResNet-18 and ResNet-50 are based on the residual network architecture. The skip connections are used to overcome the problem of vanishing gradients in deep neural networks. ResNet-18 has only 18 layers, with a simple design and high computational efficiency. It performs excellently in resource-constrained environments or lightweight applications. Liu Y et al. utilized it to develop an Infant Retinal Intelligent Diagnosis System (IRIDS) in combination with a convolutional neural network and a transformer structure. When identifying infant fundus diseases, it outperformed ophthalmologists in several key indicators (30). In the study by Nabrdalik K et al., its backbone model showed excellent accuracy in the binary classification of cardiac autonomic neuropathy (CAN) in diabetic patients (47). S V A et al. used an extended depthwise separable convolutional ResNet (DDSC-RN) with a Support Vector Machine (SVM) classifier to identify retinal biomarkers, achieving a relatively high overall accuracy (31). In contrast, ResNet-50 has 50 layers and adopts a bottleneck structure to reduce the number of parameters while maintaining high accuracy. Although S V A et al. did not directly compare it in the same kind of complex task study, when dealing with tasks that require processing complex patterns, high-resolution images, and a high level of detail, due to this structure, it has a higher accuracy than simple models. Goh JHL et al. developed a model using ResNet50 when detecting referable diabetic retinopathy (DR), providing an important reference for the overall study (32).

Swin Transformer v2 is specifically designed for computer vision. With its hierarchical feature representation and shifted window mechanism, it can efficiently capture information at different scales and has obvious advantages in processing high-resolution images, achieving remarkable results in multiple visual tasks. Li Z et al. constructed a deep learning model with it, achieving a very high average accuracy in the classification of multiple fundus diseases on two independent public datasets (33). Huang C et al. developed a Swin-MCSFNet classifier based on Swin-Transformer for retinal image quality assessment, which performed well (34).

The models of the EfficientNet series use a compound scaling method to balance the network depth, width, and input resolution, improving the performance and computational efficiency. The HDR-EfficientNet method proposed by Abbas Q et al. uses the EfficientNet-V2 network for end-to-end training to identify eye-related diseases. When evaluated on a large enhanced dataset of retinal fundus images, the average area under the curve (AUC) is outstanding (35).

Vision Transformer (ViT) introduced the Transformer architecture from natural language processing into computer vision. It divides an image into patches and operates on them just like processing text sequences. In the study by Goh JHL et al., among the four ViT models for detecting referable DR, the SWIN transformer showed significantly better AUC performance than Convolutional Neural Network (CNN) models in the tests on multiple datasets (32).

Therefore, the reason for choosing ResNet-18, ResNet-50, Swin Transformer v2, EfficientNet, and Vision Transformer (ViT) for comparison is that they each have their own characteristics in terms of network architecture, computational efficiency, feature extraction ability, and the processing of information at different scales. They can comprehensively and multi-dimensionally evaluate the performance of different types of models in the task of OCT image classification.

### 2.7 Evaluation metrics

To comprehensively assess model performance, multiple key metrics were employed for integrated analysis. First, Balanced Accuracy serves as the core criterion for measuring a model's overall classification capability in imbalanced scenarios. By calculating the average recall across all classes, it eliminates the dominance of majority classes in evaluations, fairly reflecting the model's balanced performance across categories. Second, Precision focuses on the proportion of truly positive instances among all positive predictions, a critical metric for reducing false alarm rates. Concurrently, Recall evaluates the model's ability to identify all positive samples, emphasizing its capacity to minimize missed detections. The F1-score, a harmonic mean of Precision and Recall, is specifically designed for imbalanced datasets, while AUC (Area Under the ROC Curve) measures a binary classifier's discriminative power. The ROC curve plots True Positive Rate (TPR/Recall) against False Positive Rate (FPR), and the confusion matrix provides a detailed breakdown of classification results, listing the counts of True Positives (TP), False Positives (FP), False Negatives (FN), and True Negatives (TN). This matrix enables in-depth analysis of model strengths and weaknesses across categories, guiding further optimization.

Integrating multi-dimensional metrics (accuracy, precision, recall, F1-score, AUC, and confusion matrix) ensures a comprehensive and meticulous performance analysis. This approach guarantees that developed models excel not only in overall performance but also demonstrate efficiency and reliability in handling specific classes. Such a multi-level evaluation framework lays a solid foundation for enhancing model performance.

## 3 Result

### 3.1 Ablation experiments

To validate the effectiveness of the proposed method, systematic ablation experiments were conducted to analyze classification performance under various model structure combinations. All models used identical random initialization seeds and data augmentation strategies to ensure result reliability and comparability. Using the original ResNet as the baseline model, each experimental group explored the impact of adding different layers of wavelet modules, CBAM modules, and their mixed configurations on model performance. The experimental results are shown in Table 1.

**Table 1:** Ablation study results.

Models	ACC	F1	AUC	Precision	Recal
ResNet (Original)	0.8889	0.8984	0.9867	0.9222	0.8889
ResNet + One Wavelet Convolution Layer	0.8704	0.8900	0.9809	0.9302	0.8704
ResNet + Two Wavelet Convolution Layers	0.8938	0.8892	0.9784	0.8993	0.8938
ResNet + Three Wavelet Convolution Layers	0.8933	0.8925	0.9762	0.9041	0.8933
ResNet + Four Wavelet Convolution Layers	0.8848	0.8869	0.9758	0.9028	0.8848
ResNet + One CBAM Layer	0.8860	0.8978	0.9854	0.9252	0.8860
ResNet + Two CBAM Layers	0.8904	0.8993	0.9840	0.9211	0.8904
ResNet + Three CBAM Layers	0.8973	0.8975	0.9875	0.9101	0.8973
ResNet + Four CBAM Layers	0.9005	0.9002	0.9856	0.9119	0.9005
ResNet + One Wavelet Convolution Layer + One CBAM Layer	0.8660	0.8807	0.9826	0.9156	0.8660
ResNet + Two Wavelet Convolution Layers + Two CBAM Layers	0.8918	0.8867	0.9750	0.8968	0.8918
ResNet + Three Wavelet Convolution Layers + Three CBAM Layers	0.9068	0.9129	0.9750	0.9331	0.9068
ResNet + Four Wavelet Convolution Layers + Four CBAM Layers	0.9030	0.9053	0.9795	0.9223	0.9030
ResNet (Original)	0.8889	0.8984	0.9867	0.9222	0.8889
ResNet + One Wavelet Convolution Layer	0.8704	0.8900	0.9809	0.9302	0.8704

As shown in Table 1, the standalone addition of wavelet modules yielded insignificant performance improvements, whereas incorporating CBAM modules led to a gradual performance enhancement with increasing module layers. In the mixed-configuration group, the “ResNet + three-layer wavelet + three-layer CBAM” combination achieved optimal results in accuracy, F1-score, and precision, demonstrating its significant enhancement of model classification performance and validating the effectiveness of the proposed method.

### 3.2 Statistical significance tests

Paired *t*-tests were conducted between the ResNet18 baseline model and the WARN model to systematically evaluate differences in five key classification performance metrics. Data from three independent experiments are shown in Table 2, with each experiment recording the performance of ResNet18 and WARN in accuracy (Acc), F1-score, AUC, precision, and recall.

**Table 2:** Comparative experimental results: ResNet18 vs. WARN.

Experiment no.	Model type	Acc	f1	Auc	Precision	Recall
1	ResNet18	0.888945	0.898404	0.986708	0.922182	0.888945
1	WARN	0.906843	0.912900	0.975025	0.933109	0.906843
2	ResNet18	0.891713	0.906946	0.989607	0.936012	0.891713
2	WARN	0.917391	0.920868	0.984724	0.931775	0.917391
3	ResNet18	0.892343	0.897077	0.989067	0.918552	0.892343
3	WARN	0.904606	0.907744	0.980022	0.922250	0.904606

The mean differences, *T*-statistics, and *p*-values for each metric are presented in Table 3.

**Table 3:** Statistical analysis of performance differences: mean differences, T-statistics, and *p*-values.

Metric	Average difference (enhanced - baseline)	*t*-statistic	*p*-value	Significant change
Accuracy (Acc)	+0.0186	−4.7860	0.0410	✅ Significant improvement
F1 score	+0.0130	−10.9276	0.0083	✅ Significant improvement
AUC	−0.0085	4.3130	0.0498	❌ Significant decrease
Precision	+0.0035	−0.7907	0.5120	No significant difference
Recall	+0.0186	−4.7860	0.0410	✅ Significant improvement

The WARN model demonstrated statistically significant improvements in accuracy, F1-score, and recall. Specifically, accuracy increased by an average of 1.86% (*p* = 0.041), F1-score by 1.30% (*p* = 0.0083), and recall by 1.86% (*p* = 0.041), indicating that the wavelet feature extraction and CBAM attention mechanism effectively enhanced the model's ability to identify positive samples and improved overall classification performance. However, the AUC metric showed a minor 0.85% decrease (*p* = 0.0498), a statistically significant change suggesting potential reductions in discriminant stability across different classification thresholds. Although precision increased by 0.35%, the *p*-value of 0.512 did not reach statistical significance, indicating unclear effectiveness in reducing false positives.

Pooling data from three experiments, WARN consistently outperformed the ResNet18 baseline model, particularly in identification capability and overall accuracy. While the enhancement strategy positively impacted key performance metrics, the decrease in AUC highlights a need to monitor discriminant stability in practical applications, which could be addressed through further optimization to balance metric performance.

### 3.3 Experimental results

**Figure 6:**
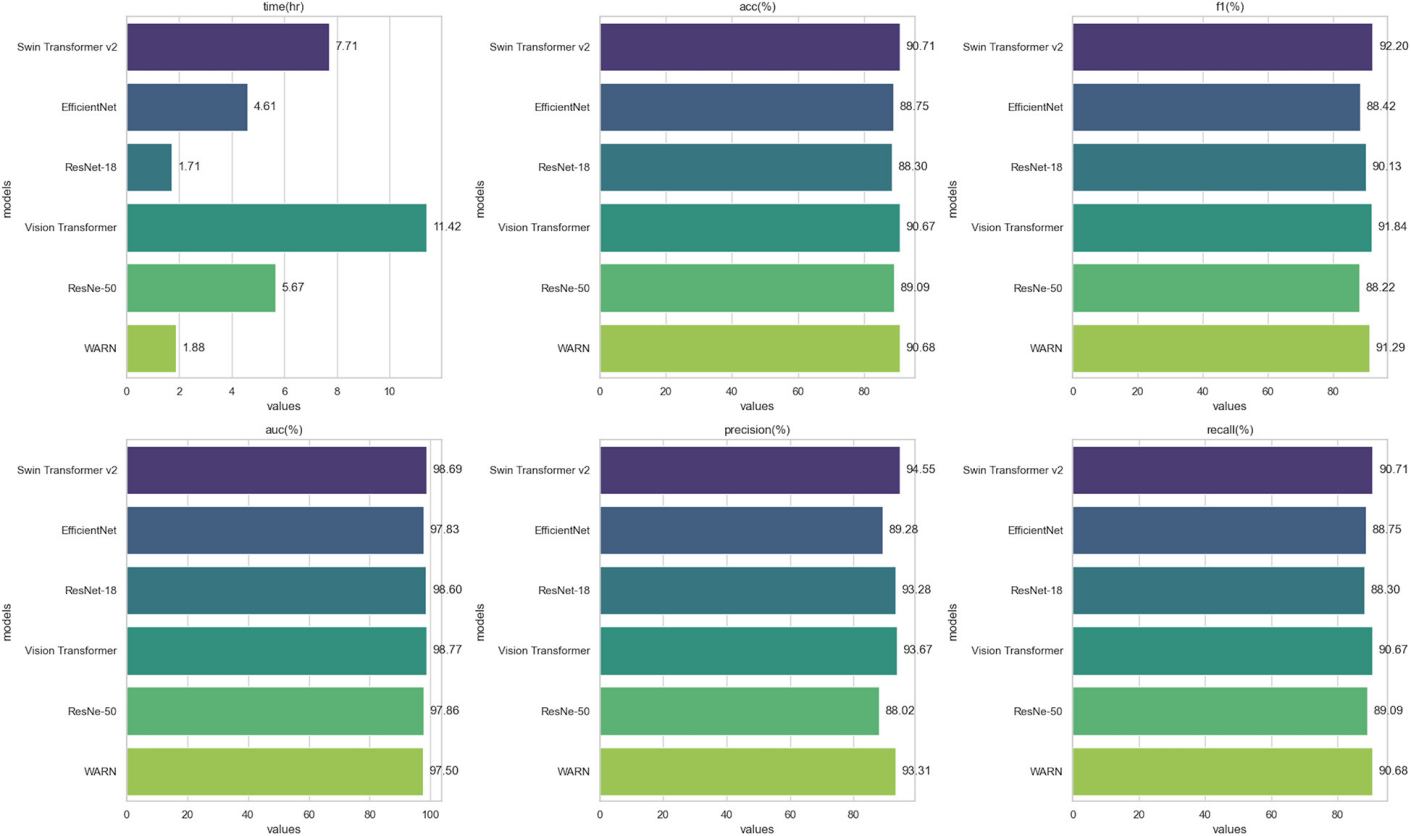
Experimental results of ResNet-18, ResNet-50, Swin Transformer v2, EfficientNet, Vision Transformer, and WARN.

To systematically assess the classification efficiency of different network architectures, comprehensive metric tests were conducted on ResNet-18, ResNet-50, Swin Transformer v2, EfficientNet, Vision Transformer (ViT), and the WARN model. Table 4 details the runtime, accuracy (Acc), F1-score, AUC, precision, and recall of each model on the test set, while Figure 6 visually presents the horizontal comparison trends of core metrics.

**Table 4:** Experimental results of ResNet-18, ResNet-50, Swin Transformer v2, EfficientNet, vision transformer, and WARN.

Models	Time (hr)	Acc (%)	f1 (%)	Auc (%)	Precision (%)	Recall (%)
ResNet-18	1.711	88.89%	89.84%	98.67%	92.22%	88.89%
ResNe-50	5.666	89.09%	88.22%	97.86%	88.02%	89.09%
Swin Transformer v2	7.707	90.71%	92.20%	98.69%	94.55%	90.71%
EfficientNet	4.609	88.75%	88.42%	97.83%	89.28%	88.75%
Vision Transformer	11.42	90.67%	91.84%	98.77%	93.67%	90.67%
WARN	1.881	90.68%	91.29%	97.50%	93.31%	90.68%

The table presents runtime and classification performance metrics for six deep learning models. In terms of runtime, ResNet-18 was the fastest (1.711 h), while Vision Transformer (ViT) was the slowest (11.42 h). Swin Transformer v2 and ViT demonstrated superior performance across multiple metrics: Swin Transformer v2 achieved the highest accuracy (90.71%) and F1-score (92.20%), whereas ViT ranked first in AUC (98.77%). The WARN model showed a shorter runtime (1.881 h) alongside high accuracy (90.68%), F1-score (91.29%), and recall (90.68%), demonstrating an optimal balance between performance and efficiency. ResNet-50 and EfficientNet exhibited relatively lower metrics, with ResNet-50 having the lowest F1-score (88.22%) and EfficientNet showing slightly lower accuracy (88.75%) compared to other models.

**Figure 7:**
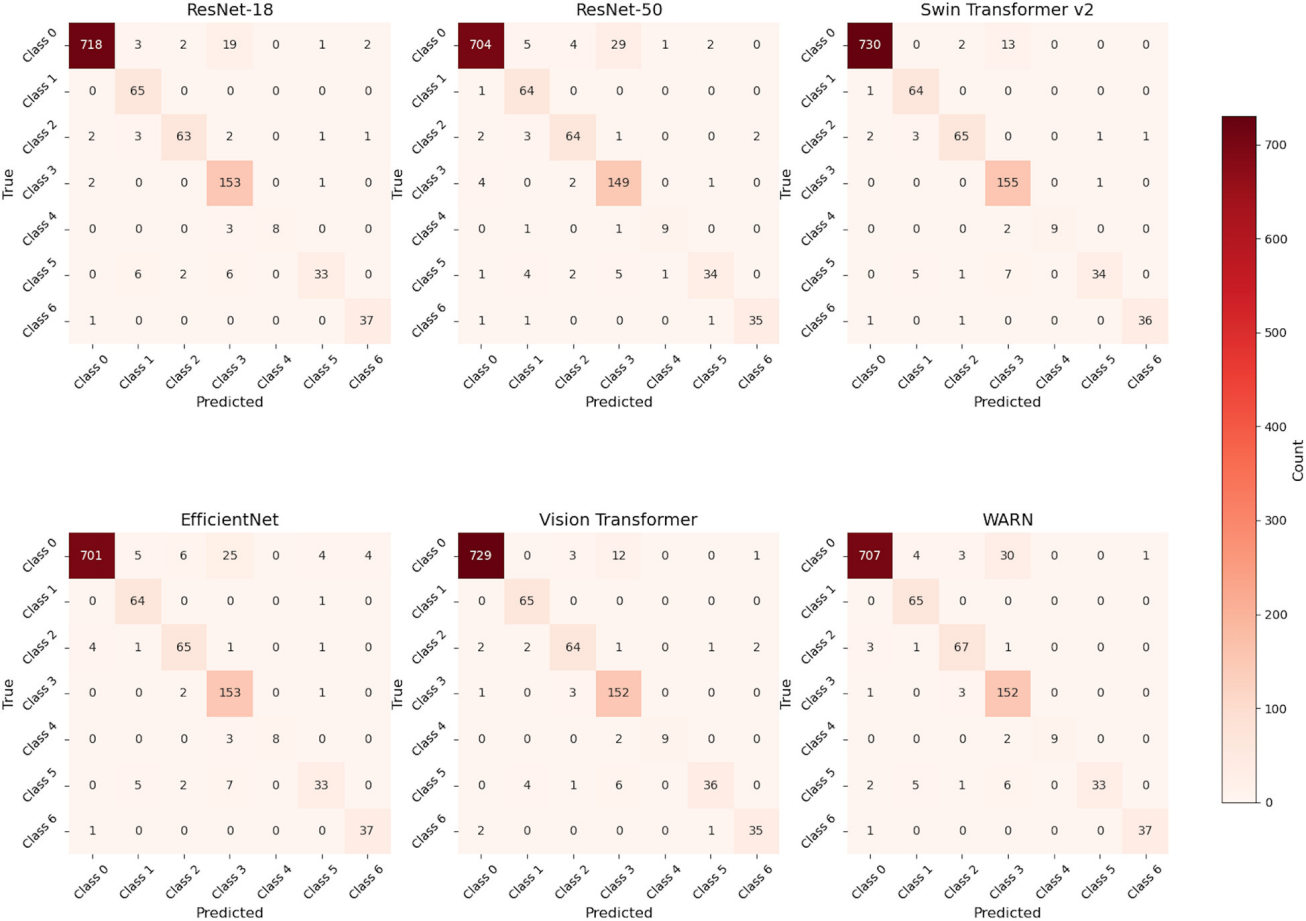
Confusion matrices of ResNet-18, ResNet-50, Swin Transformer v2, EfficientNet, Vision Transformer, and WARN.

Overall, Swin Transformer v2 and ViT excelled in performance, while WARN stood out for its high performance and fast runtime, making it an efficient and accurate choice. Furthermore, a detailed analysis of the confusion matrix in Figure 7 allows for further identification of specific misclassification patterns by examining the frequency of predictions across categories.

To assess the classification performance of different vision models in retinal diseases, three core metrics—Precision, Recall, and F1-Score—were calculated based on confusion matrices. The experiment involved six models [ResNet-18, ResNet-50, Swin Transformer v2, EfficientNet, Vision Transformer (ViT), and WARN] for classifying seven retinal diseases, including age-related macular degeneration (AMD) and diabetic macular edema (DME). The table below presents detailed evaluation results for each model across disease categories, reflecting their identification accuracy, positive sample capture capability, and comprehensive balance performance. Specific findings are shown in Table 5.

**Table 5:** The precision, recall, and F1-score of ResNet-18, ResNet-50, Swin Transformer v2, EfficientNet, vision transformer, and WARN across various disease categories.

Models	Precision	Recall	F1-Score
AMD	ResNet-18	0.9931	0.9638
	ResNet-50	0.9874	0.9450
	Swin v2	0.9946	0.9799
	EfficientNet	0.9929	0.9409
	Vision Transformer	0.9932	0.9785
	WARN	0.9902	0.9490
DME	ResNet-18	0.8442	1.0000
	ResNet-50	0.8205	0.9846
	Swin v2	0.8889	0.9846
	EfficientNet	0.8533	0.9846
	Vision Transformer	0.9155	1.0000
	WARN	0.8667	1.0000
ERM	ResNet-18	0.9403	0.8750
	ResNet-50	0.8889	0.8889
	Swin v2	0.9420	0.9028
	EfficientNet	0.8667	0.9028
	Vision Transformer	0.9014	0.8889
	WARN	0.9054	0.9306
NO	ResNet-18	0.8361	0.9808
	ResNet-50	0.8054	0.9551
	Swin v2	0.8757	0.9936
	EfficientNet	0.8095	0.9808
	Vision Transformer	0.8786	0.9744
	WARN	0.7958	0.9744
RAO	ResNet-18	1.0000	0.7273
	ResNet-50	0.8182	0.8182
	Swin v2	1.0000	0.8182
	EfficientNet	1.0000	0.7273
	Vision Transformer	1.0000	0.8182
	WARN	1.0000	0.8182
RVO	ResNet-18	0.9167	0.7021
	ResNet-50	0.8947	0.7234
	Swin v2	0.9444	0.7234
	EfficientNet	0.8250	0.7021
	Vision Transformer	0.9474	0.7660
	WARN	1.0000	0.7021
VID	ResNet-18	0.9250	0.9737
	ResNet-50	0.9459	0.9211
	Swin v2	0.9730	0.9474
	EfficientNet	0.9024	0.9737
	Vision Transformer	0.9211	0.9211
	WARN	0.9737	0.9737

After evaluating the classification performance across multiple retinal disease categories, significant differences in model performance were observed. Overall, Swin Transformer v2 and Vision Transformer (ViT) demonstrated superior performance in Precision, Recall, and F1-Score, particularly in classifying AMD, DME, and VID. In contrast, ResNet-50 and EfficientNet showed moderate to weak performance in most tasks, possibly constrained by model complexity or feature extraction capability.

The proposed WARN method exhibited excellent comprehensive performance across key metrics. Notably, it achieved Precision, Recall, and F1-Score of 0.9737 for VID, highlighting strong discriminative ability. For DME and RAO, WARN's Recall approached or reached perfect scores, indicating exceptional positive sample identification—critical for clinical scenarios demanding low missed diagnosis rates. In RVO classification, while WARN's Recall was relatively low (0.7021), its perfect Precision (1.0) ensured highly reliable predictions, making it suitable for tasks intolerant of misdiagnosis. However, WARN showed notably lower Precision (0.7958) for the normal (NO) category, revealing a tendency to misclassify abnormal images as normal. This underscores the need for future optimization, such as enhancing normal sample learning or introducing stronger discriminative mechanisms.

In summary, WARN demonstrates robust and competitive performance across diverse disease classifications, excelling in Precision and select Recall metrics, which highlights its potential as an auxiliary diagnostic tool. However, improvements are needed to balance Recall and Precision and enhance normal category identification.

### 3.4 Performance validation — based on retinal disease data from Shanxi Eye Hospital

**Figure 8:**
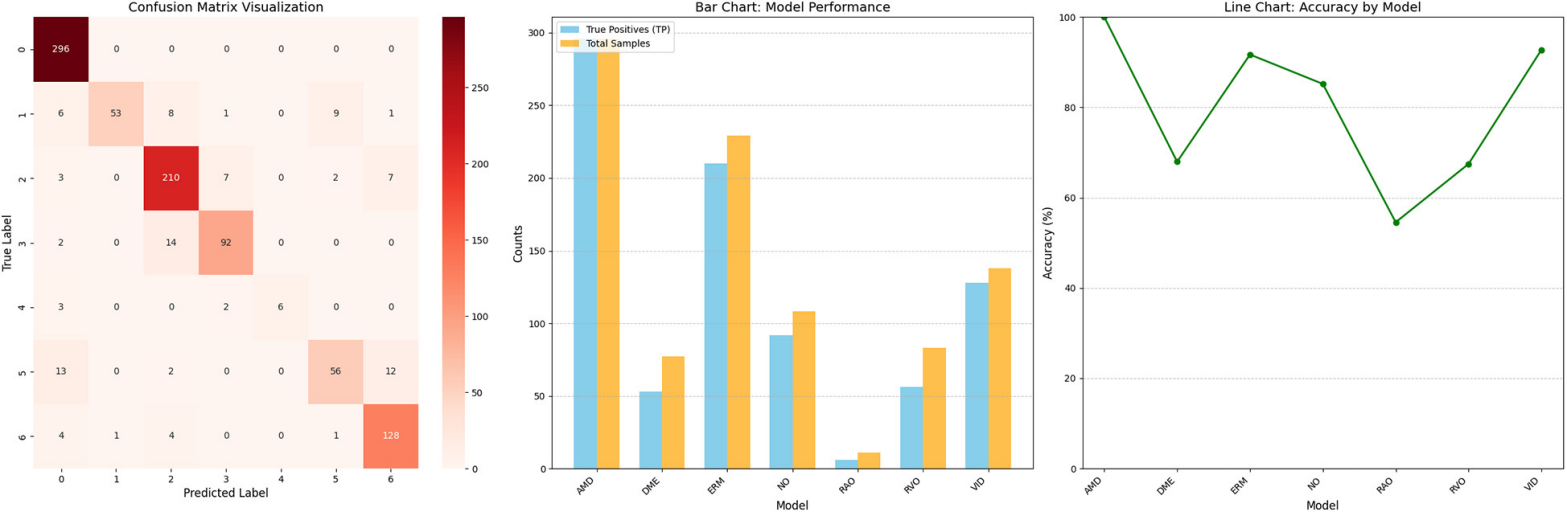
Analysis of WARN's performance based on the dataset from Shanxi Eye Hospital: confusion matrix, correct classification counts, and accuracy rates by category.

 Table 6 presents the model's performance across different retinal disease classifications using the dataset provided by the Shanxi Eye Hospital. Figure 8 illustrates the WARN model's performance analysis based on the Shanxi Eye Hospital dataset, including a confusion matrix, the number of correctly classified samples, and the accuracy rate for each category.

**Table 6:** Analysis of WARN's performance based on the dataset from Shanxi Eye Hospital: confusion matrix, correct classification counts, and accuracy rates by category.

Models	AMD accuracy (%)	DME accuracy (%)	ERM accuracy (%)	NO accuracy (%)	RAO accuracy (%)	RVO accuracy (%)	VID accuracy (%)
True Positives	296	53	210	92	6	56	128
Total Samples	296	77	229	108	11	83	138
WARN	100	67.95	91.70%	85.19	54.55	67.47	92.75

First, in the age-related macular degeneration (AMD) category, there were 296 total samples, all of which were correctly identified as true positives, resulting in a 100% accuracy rate for the WARN model in this category. Next, the diabetic macular edema (DME) category included 77 samples, of which 53 were correctly identified as true positives, yielding an accuracy rate of 67.95% for the WARN model. In the epiretinal membrane (ERM) category, with 229 total samples, 210 were correctly identified as true positives, leading to a WARN model accuracy of 91.70%. For the normal fundus image (NO) category, there were 108 samples, 92 of which were correctly identified, resulting in an 85.19% accuracy rate for the WARN model. The retinal artery occlusion (RAO) category had 11 samples, 6 of which were correctly identified, giving the WARN model an accuracy rate of 54.55%. The retinal vein occlusion (RVO) category was based on 83 samples, 56 of which were correctly identified, resulting in a 67.47% accuracy rate for the WARN model. Finally, in the vitreomacular interface disease (VID) category, 128 out of 138 total samples were correctly identified as true positives, yielding a WARN model accuracy rate of 92.75%. These data comprehensively detail the WARN model's performance in various retinal disease classifications.

### 3.5 Grad-CAM

**Figure 9:**
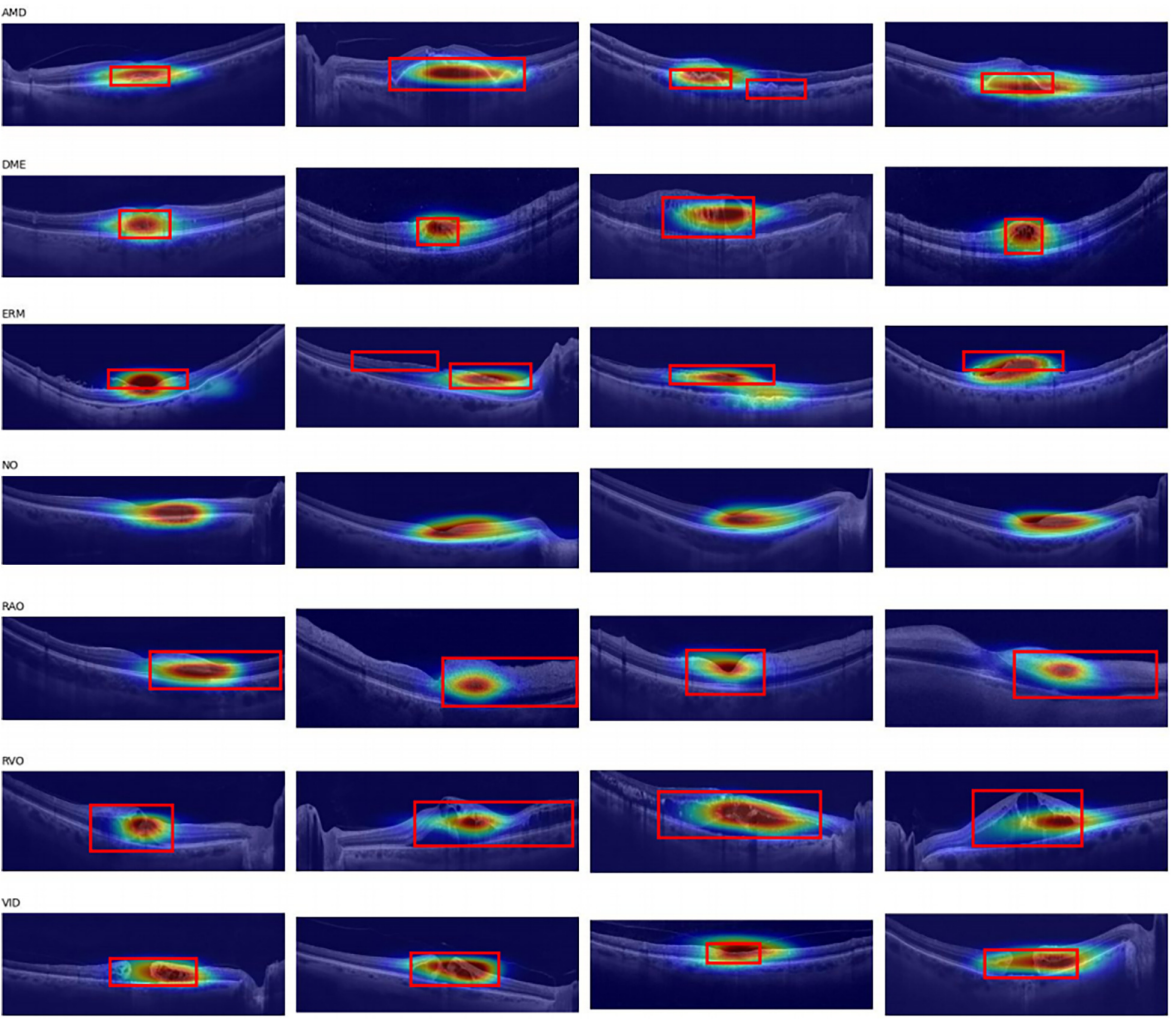
Employing Grad-CAM on the OCTDL test Set.

To gain a deeper understanding of the model's decision-making process and validate its reliability in practical applications, we employed Grad-CAM (Gradient-weighted Class Activation Mapping) technique. As an effective visualization method, Grad-CAM generates heatmaps by calculating the gradients of the target class with respect to the feature maps output by the convolutional layers, thereby intuitively demonstrating the regions of the image that the model focuses on when making predictions. Specifically in our study, the last layer of the WARN model was selected as the target layer for Grad-CAM. By applying the Grad-CAM technique to the images in the test set, we generated corresponding heatmaps, which are presented in Figure 9. These heatmaps clearly reveal the model's focus on lesions of different types of retinal diseases. We overall framed the retinal areas with lesions on the images. From the images, it can be seen that the model pays high attention to the lesion areas.

## 4 Discussion

This study integrates the CBAM attention mechanism and wavelet convolution based on ResNet to construct a network structure named WARN. Through ablation experiments, significance tests, and comparative experiments, the effectiveness and performance advantages of the proposed model are systematically verified. Compared with previous studies, this work has the following prominent features: First, it is committed to achieving single-shot synchronous classification of seven retinal diseases, significantly improving diagnostic efficiency; second, it uses the latest public OCT image dataset for training, ensuring the model's generalization ability and cutting-edge nature; in addition, it still achieves excellent classification performance with a short training time, demonstrating good practical value. Finally, the model was tested and analyzed on a private dataset provided by the Shanxi Eye Hospital, further verifying its applicability in actual clinical scenarios.

Compared with previous studies, in terms of the complexity of the classification task, most existing studies only focus on the classification of single diseases or up to three to four types of retinal diseases (21, 36–38), while the WARN model proposed in this paper is committed to achieving single-shot synchronous classification of seven common retinal diseases, greatly improving clinical diagnostic efficiency and practicality. Second, in terms of network structure design, existing methods mostly adopt standard convolutional neural network structures, such as VGG, ResNet, or their lightweight variants (20, 39–41), while this paper integrates the CBAM attention mechanism and wavelet convolution module based on ResNet. The introduction of CBAM enables the model to focus on the lesion area and enhance key feature expression (42), while wavelet convolution enhances the multi-scale texture modeling ability of images, helping to capture subtle pathological changes in OCT images, thereby improving overall performance without significantly increasing the number of parameters. Third, in terms of data usage and generalization ability, this paper uses the latest public OCT image dataset for training. Compared with the use of specific datasets in early work (19), it ensures the model's cutting-edge nature and generalization ability. In addition, in terms of model efficiency and practical value, although many studies have achieved high classification accuracy (43, 44), they often rely on long training times or complex network structures, limiting their practical deployment possibilities; while this paper achieves excellent performance in a short training time and has good engineering implementation potential. Finally, in terms of clinical applicability verification, different from most studies that only evaluate model performance based on public datasets, this paper further tests and analyzes the model on a private dataset provided by the Shanxi Eye Hospital, verifying its stability and reliability in real clinical scenarios. In summary, WARN performs well in classification granularity, network structure, training efficiency, and clinical adaptability, providing an efficient solution for intelligent auxiliary diagnosis of retinal diseases.

### 4.1 Discussion on ablation experiments

To verify the effectiveness of the proposed method, we conducted systematic ablation experiments to compare the classification performance under various model structure combinations. Using the original ResNet as the baseline model, it achieved an accuracy (ACC) of 0.8889, an F1 score of 0.8984, and an AUC value of 0.9867, demonstrating good overall classification ability. On this basis, wavelet transform modules and CBAM attention mechanisms were introduced respectively for comparative experiments. The results show that the wavelet module improves the model's ability to capture image detail features to a certain extent, with the three-layer wavelet structure performing best (ACC = 0.8933, F1 = 0.8925), but performance tends to decline as the number of layers increases, possibly due to redundant information introduction or overfitting. In contrast, the CBAM module significantly improves model performance, especially showing more stable gain effects when stacking multiple layers. The three-layer CBAM structure achieves the optimal AUC value (0.9875), while the four-layer CBAM performs best in ACC (0.9005) and F1 (0.9002), indicating that CBAM can effectively enhance the model's ability to focus on key features.

To further explore the synergistic effects between different modules, we also tested the hybrid structure of wavelet and CBAM. The results show that the “ResNet + three-layer wavelet + three-layer CBAM” combination exhibits the strongest comprehensive performance: accuracy reaches 0.9068, F1 score is as high as 0.9129, precision is 0.9331, and recall is 0.9068. Although its AUC value (0.9750) is slightly lower than that of the pure CBAM model, it outperforms all other configurations in the remaining core indicators, demonstrating the complementary advantages of combining wavelet transform and CBAM attention mechanism in feature extraction and discriminative ability. The effectiveness of the fusion strategy is verified through systematic experiments, and the joint structure of “three-layer wavelet + three-layer CBAM” is confirmed as a promising improvement scheme, so this fusion strategy is selected in subsequent experiments.

### 4.2 Discussion on significance experiments

By performing paired t-tests between the ResNet18 baseline model and WARN, we systematically evaluated their differences in five key classification performance indicators. The results show that the enhanced model exhibits statistically significant improvements in accuracy (Acc), F1 score, and recall (Recall). Specifically, the accuracy increased by an average of 1.86% (*p* = 0.041), the F1 score by 1.30% (*p* = 0.0083), and the recall by 1.86% (*p* = 0.041). These results indicate that the introduced wavelet feature extraction and CBAM attention mechanism effectively enhance the model's ability to recognize positive samples, thereby improving overall classification performance.

To comprehensively evaluate the performance of WARN in retinal disease classification, we conducted comparative experiments with several current mainstream deep learning models, including ResNet-18, ResNet-50, Swin Transformer v2, EfficientNet, and Vision Transformer (ViT). Evaluation metrics cover runtime (training duration), accuracy (acc%), F1 score (f1%), AUC value (auc%), precision (precision%), and recall (recall%), with all results validated on the same test set to ensure fairness and comparability. In terms of operational efficiency, WARN only requires 1.881 h to complete the training process, slightly higher than ResNet-18 (1.711 h) but significantly better than other complex models such as Swin Transformer v2 (7.707 h) and Vision Transformer (11.42 h). This advantage makes WARN more suitable for practical deployment and clinical applications, especially in resource-constrained or fast-response scenarios.

In terms of classification performance, WARN demonstrates promising results: accuracy (acc) is 90.68%, second only to Swin Transformer v2 (90.71%) and superior to ResNet-50 (89.09%), ResNet-18 (88.89%), and EfficientNet (88.75%); the F1 score is 91.29%, ranking third among all models, slightly lower than Swin Transformer v2 (92.20%) and Vision Transformer (91.84%), indicating its capability to balance precision and recall; precision is 93.31%, second only to Swin Transformer v2 (94.55%), demonstrating high reliability in predicting positive samples; recall is 90.68%, on par with Swin Transformer v2 and significantly better than ResNet-18 (88.89%) and EfficientNet (88.75%), indicating the model's effectiveness in identifying more true positive cases; the AUC value is 97.50%, slightly lower than Vision Transformer (98.77%), Swin Transformer v2 (98.69%), and ResNet-18 (98.67%), but still maintaining strong classification discriminative ability while ensuring efficient training speed.

Comprehensively, although Swin Transformer v2 has slight advantages in indicators such as accuracy, F1 score, and AUC, its high computational cost limits practicality; Vision Transformer, despite the best AUC performance, requires 11 h of training, making it difficult to meet real-time or low-latency requirements. In contrast, WARN achieves high-level performance in all metrics while maintaining low training overhead, reflecting its excellent balance between accuracy and efficiency.

Furthermore, by introducing the wavelet transform and CBAM attention mechanism, WARN can better capture texture details and lesion boundary information in retinal images, thereby enhancing the ability to identify early minor lesions. As a deep learning model integrating wavelet convolution, residual connections, and attention mechanisms, WARN demonstrates superior comprehensive performance in retinal disease classification tasks. It not only outperforms or approaches existing mainstream models in multiple key evaluation indicators but also has significant advantages in training efficiency.

### 4.3 Discussion on confusion matrix

Through the detailed analysis of the confusion matrix in Figure 7, we can further understand the number of times each category is predicted into various categories, thereby identifying specific types of misclassification issues. WARN shows significant improvements in specific categories (such as ERM and RAO), reflecting the effectiveness of CBAM and wavelet convolution modules—CBAM focuses on key areas through spatial and channel attention, while wavelet convolution expands the receptive field and enhances the capture of local texture and global structure (especially in categories with rich lesion details like ERM and VID). First, in ERM (epiretinal membrane) detection, recall increased from 87.50% in ResNet-18 to 93.06%, a 5.56 percentage point improvement, and the F1 score also rose from 90.68% to 91.78%. This improvement benefits from the wavelet convolution module's more effective capture of local details (such as membranous structures) and the CBAM attention mechanism's weighted optimization of feature maps, reducing confusion with normal samples (NO). Analysis of the confusion matrix shows a significant reduction in misdiagnosis: ResNet-18 had 9 false negatives (FN), while WARN only had 5 FN. In the RAO (retinal artery occlusion) task, due to the scarcity of such samples (only 11 cases), they are easily misdiagnosed as normal samples. However, WARN enhances the ability to extract vascular occlusion features by introducing wavelet convolution, increasing recall from 72.73% to 81.82%, reaching levels comparable to Swin and ViT, and the F1 score leaped from 84.21% to 90.00%. Meanwhile, the number of false negatives decreased from 3 to 2, further verifying the model's enhanced sensitivity to minority categories. For VID (vitreomacular interface disease), although ResNet-18 already had a high recall (97.37%), WARN maintained the same recall while increasing the F1 score from 95.95% to 97.37%. This primarily attributes to the CBAM attention mechanism's priority processing of global features (such as macular traction), effectively reducing the false negative rate. Additionally, while recall rates for AMD and NO categories slightly decreased (96.44%→94.90% and 98.08%→97.44%, respectively), F1 scores remained at high levels (AMD: 97.06%; NO: 86.86%), indicating that the model pays more attention to minority categories in resource allocation, and overall classification performance remains competitive. DME and RVO showed stable performance, with DME maintaining a 100% recall rate. Overall, compared with ResNet-18, WARN excels in key disease categories such as ERM, RAO, and VID, reflecting CBAM's ability to reasonably allocate attention weights and wavelet convolution's advantages in expanding the receptive field and capturing lesion details. The improvement in F1 score indicates that the model is more robust and reliable in clinical practical applications.

### 4.4 Discussion on hospital experiment results

Using the constructed WARN to test the dataset provided by Shanxi Eye Hospital, the test results are shown in Table 6 and Figure 8, with an accuracy of 89.18%, precision of 79.94%, and recall of 90.85%. For age-related macular degeneration (AMD), the model achieved 100% accuracy, with both the number of true positives (TP) and total samples being 296, indicating the model's excellent recognition ability in this category with almost no false positives. Analysis of misclassification cases in the test set reveals that some DME images were incorrectly classified as AMD (age-related macular degeneration) or ERM (epiretinal membrane), mainly because the lesion features of DME and AMD are very similar in some cases, especially in macular region changes, making it difficult for the model to distinguish between the two diseases. Additionally, some images contain not only DME lesions but also other types of lesions such as ERM, further increasing the complexity of classification. Since these images have multiple lesion features simultaneously, the model may tend to select more obvious or prominent feature categories for classification, leading to errors. For the epiretinal membrane (ERM) category, the model demonstrated high-level accuracy at 91.70%, with TP = 210 and total samples = 229, indicating significant effectiveness in handling this category. The classification accuracy for normal (NO) images was 85.19%, with TP = 92 and total samples = 108, showing good performance but still with room for improvement. For the RAO (retinal artery occlusion) and RVO (retinal vein occlusion) categories, the model performed relatively poorly, with accuracies of 54.55% and 67.47%, respectively. In-depth analysis concludes that one main reason is insufficient sample quantity, which is clearly inadequate for the model to fully learn the features of these diseases. The small sample size directly limits the model's learning ability, causing poor performance when facing new, unseen data. Additionally, the huge difference in sample quantity between different categories in the dataset exacerbates the class imbalance problem, possibly leading the model to pay more attention to categories with more samples during training while ignoring the less-sampled RAO and RVO. Finally, in the vitreomacular interface (VID) category, the model also performed excellently, with an accuracy of 92.75%, TP = 128, and total samples = 138. Through this series of test results, we fully verified the effectiveness and reliability of the constructed network model.

### 4.5 Limitations and future directions

The application of AI-assisted diagnostic systems in ophthalmic clinical practice holds immense promise. By leveraging deep learning to automatically analyze OCT images, AI can help clinicians rapidly diagnosis for multiple retinal diseases, thereby improving diagnostic efficiency and reducing the incidence of misdiagnosis and missed diagnosis. Especially for dynamic lesions requiring long-term monitoring, such as different stages of retinal vein occlusion, AI can provide continuous and consistent assessment results to assist doctors in formulating treatment plans. Additionally, AI-assisted diagnosis contributes to scientific basis for personalized treatment regimens, enhancing patient management and therapeutic outcomes.

Despite the achievements of this study, several limitations need to be addressed. First, the dataset size is limited, covering only seven common retinal diseases, which undoubtedly restricts the model's application scope. Second, insufficient data diversity is a notable issue—for example, uneven ethnic distribution in samples and single OCT device models—factors that may affect the model's universality and accuracy. Meanwhile, inadequate dataset samples and the presence of multiple lesion types in single OCT images impose higher requirements on accurate annotation and model training, potentially leading to incomplete model learning or bias. These issues highlight the need for practical improvements in future research to enhance model robustness and applicability.

To further improve the diagnostic performance of AI models, a potential direction is integrating multimodal data, such as optical coherence tomography angiography (OCTA). Notably, OCTA provides detailed information on retinal microcirculation, which is crucial for the diagnosis and differentiation of various retinal diseases. Additionally, constructing multimodal models by incorporating clinical metadata (e.g., disease stage) can further enhance classification accuracy (45). By integrating multimodal data, we can obtain more comprehensive pathological information to improve diagnostic accuracy. Furthermore, developing real-time AI systems and seamlessly integrating them into existing clinical workflows (e.g., PACS systems) represents an important future direction. Such systems can not only provide clinicians with immediate diagnostic suggestions but also optimize medical resource allocation, further promoting the practical application of AI in ophthalmology. In follow-up studies, expanding sample sizes through multicenter collaboration—employing federated learning techniques to allow distributed parameter updates for joint model training without sharing raw data—can reduce class imbalance and enhance the model's ability to learn features of various diseases (46). Through continuous technological innovation and interdisciplinary collaboration, we eagerly anticipate AI playing a more significant role in the diagnosis and treatment of retinal diseases in the future.

## 5 Conclusion

This study developed and validated a deep-learning algorithm, WaveAttention-ResNet (WARN), to enhance the classification accuracy of seven common retinal diseases in optical coherence tomography (OCT) images. By integrating wavelet convolution to expand the model's receptive field and the CBAM attention mechanism to precisely allocate attention weights across spatial and channel dimensions of OCT images, WARN enables effective capture of both local and global features. Experimental results demonstrate that in the public OCTDL dataset, ablation experiments and significance tests confirmed WARN's effectiveness: it achieved 90.68% accuracy, 91.29% F1 score, 97.50% AUC, 93.31% precision, and 90.68% recall with relatively short training time. Additionally, in an independent test dataset from Shanxi Eye Hospital, the model performed robustly, yielding 89.18% accuracy, 90.85% recall, and 79.94% precision. These findings validate WARN's efficiency and feasibility for retinal disease classification. More importantly, this research highlights the non-negligible value and broad prospects of AI technology in auxiliary medical diagnosis. Future work will further optimize model performance and explore its applications in broader medical image analysis tasks to advance diagnostic efficiency.

## Data Availability

The datasets presented in this article are not readily available because the data supporting the findings of this study are available upon reasonable request. Interested researchers should contact zhanglj2004@163.com to submit requests. Requests to access the datasets should be directed to zhanglj2004@163.com.
